# Delayed Diagnosis in a Rare Case of Pulmonary Sarcoidosis Presenting as Unilateral Hilar Lymphadenopathy and Fever of Unknown Origin

**DOI:** 10.7759/cureus.15792

**Published:** 2021-06-21

**Authors:** Pravin M Thomas, Tarig Mabrouk, Yiting Li, Sara L Wallach

**Affiliations:** 1 Internal Medicine, St. Francis Medical Center, Trenton, USA; 2 Internal Medicine, Hackensack Meridian School of Medicine, Nutley, USA

**Keywords:** sarcoidosis, unilateral hilar lymphadenopathy, fever of unknown origin

## Abstract

In this report, we present a rare case of sarcoidosis presenting as fever of unknown origin with unilateral hilar lymphadenopathy, in contrast with the typical presentation of sarcoidosis. Sarcoidosis presenting with asymmetric or isolated unilateral lymphadenopathy is highly unusual. Sarcoidosis is also an uncommon cause of fever of unknown origin. This atypical presentation led to diagnostic delays in our case. This case report emphasizes the importance of considering sarcoidosis early in the differential diagnosis of fever of unknown origin with unilateral hilar lymphadenopathy.

## Introduction

We report a diagnostically challenging case of fever of unknown origin in a patient with unilateral hilar lymphadenopathy and blepharitis who was eventually diagnosed with sarcoidosis on biopsy. Sarcoidosis with unilateral hilar lymphadenopathy can present a serious diagnostical dilemma. Developing a familiarity with the various atypical presentations of sarcoidosis can prevent delays in its diagnosis and management.

An abstract based on this case was presented as a virtual poster at the American Thoracic Society on May 13, 2021.

## Case presentation

Our patient was a 48-year-old female with a past medical history of hyperlipidemia, hypertension, and gastroesophageal reflux disease (GERD) who presented to the hospital with a two-week history of dry cough, fever, red conjunctiva, chills, malaise, myalgias, and generalized weakness. She reported a weight loss of 15 pounds in the past year. She had been a smoker but had quit five years prior to the presentation. Prior to this hospital visit, she had consulted an outpatient clinic with complaints of fever, chills, and cough. She had received a flu vaccine a month prior. She had been thought to have a viral respiratory infection as an outpatient. Ibuprofen, and later azithromycin, had been prescribed, which had not alleviated her initial symptoms before the current presentation.

On physical exam, she had a temperature of 39.3 °C in the emergency department. She was tachycardic at 130 beats per minute and tachypneic with a respiratory rate of 24 breaths per minute. Her blood pressure was 149/74 mmHg. She had bilateral conjunctival injection, but her eyelids were grossly unremarkable. There was left axillary lymphadenopathy. A physical breast exam was normal with no palpable masses. There was no other palpable adenopathy, skin rashes, hepatomegaly, splenomegaly, clubbing, or edema. The respiratory and cardiovascular exams were otherwise unremarkable. Her ECG showed sinus tachycardia at 106 beats per minute but was otherwise unremarkable.

Relevant laboratory workup included a leukocytosis of 16.7 Thou/uL (normal range: 3.8-10.2) with a neutrophilic predominance of 82.9%. She had chronic anemia with a hemoglobin (Hb) level of 8.9 g/dl (11.9-15.1) and a mean corpuscular volume (MCV) of 76 fL (78.2-98.4). Her ferritin was more than 7,500 ng/ml (11-307), with a total iron-binding capacity of 204 (250-450), and a low iron level of 37 ug/dl (50-212). Her angiotensin-converting enzyme (ACE) level was 97 U/l (8-52). Her C-reactive protein (CRP) was 19.05 mg/dl (0.02-1.00), D-dimer was 13.46 ugFEU/ml (<0.50), aspartate aminotransferase (AST) was 54 U/L (13-39), and alanine aminotransferase (ALT) was 66 U/L (7-52); she had calcium of 9.0 mg/dl (8.6-10.2), albumin of 3.0 g/dl (3.5-5.0), corrected calcium of 9.8 mg/dl, serum 25-hydroxy vitamin D level of 22.4 ng/ml (30-100), creatinine of 1.11 mg/dl (0.49-1.01), and lactate dehydrogenase (LDH) of 344 U/L (140-271).

**Figure 1:**
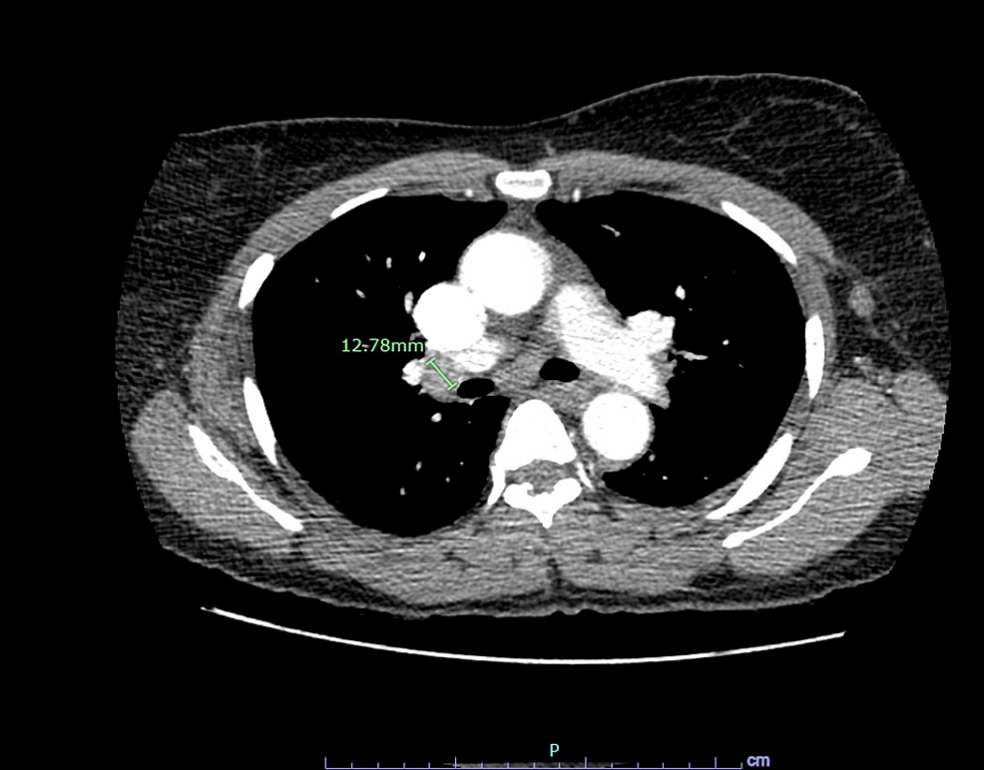
CTA showing right hilar lymphadenopathy of 1.3 cm CTA: computed tomography angiography

**Figure 2:**
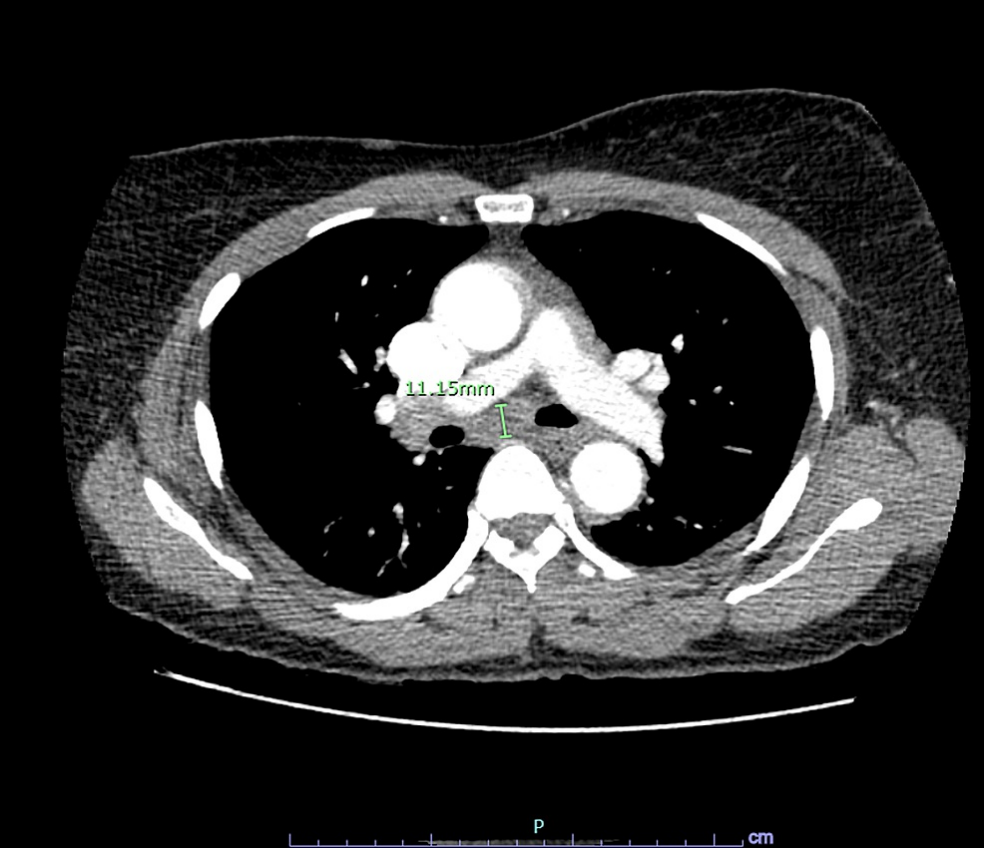
CTA image of lymphadenopathy near the carina of 1.1 cm CTA: computed tomography angiography

The patient had not undergone a prior mammogram. A CT angiography (CTA) was performed, which ruled out any pulmonary embolus. There was no evidence of a pulmonary embolus, or pulmonary nodules, or infiltrates. However, the CTA revealed right hilar lymphadenopathy of 1.3 cm in size and subcarinal lymphadenopathy of 1.1 cm (Figures 1, 2).

A CT of the abdomen revealed a cystic mass of the left retroperitoneum measuring 14 x 11 x 6 cm. The official radiology report suggested that it was of a benign etiology, which could include cystic lymphangioma, mesenteric cyst, or duplication cyst. A cystic neoplasm was considered unlikely in the radiology report. The patient was placed on broad-spectrum antibiotics, including vancomycin, cefepime, metronidazole, and azithromycin as well as intravenous fluids to treat a possible unknown infective source while awaiting cultures and further investigation. It was initially thought that a respiratory or abdominal source was the possible infective foci, which guided the selection of antibiotics.

The infectious disease team suspected an intrabdominal or lung infective etiology. An initial differential by the infectious diseases specialists, aside from infection, also included lymphoma and autoimmune processes such as sarcoidosis. The pulmonology team later advised that there was no evidence of pneumonia or tracheobronchitis. The hematology team emphasized the need to rule out lymphoma. Blood cultures, sputum cultures, and urine cultures were all negative. Antibiotics were initially tapered to ceftriaxone and metronidazole and then completely discontinued on day six. Drainage of the abdominal cyst revealed 250 mL of pale straw-colored serous fluid. Fluid chemistries were negative for an infectious or malignant etiology.

The patient underwent extensive investigation. Serum thyroid-stimulating hormone (TSH) levels, serum creatine kinase levels, serum HIV tests, rapid plasma reagin (RPR) test, an acute hepatitis panel, three acid-fast sputum stains, QuantiFERON®-TB Gold test, anti-SS-A antibody levels, anti-SS-B antibody levels, and urinalysis were all normal. She did, however, test positive for an antinuclear antibody (ANA) speckled+ pattern. The ANA titer was 1:320. The double-stranded DNA antibody levels were within the normal range at 49 Iu/ml (<200 Iu/ml). A bone marrow biopsy of the iliac crest showed no evidence of malignancy or acid-fast bacilli.

Surgery was consulted for an excisional lymph node biopsy of the left axillary lymph node. The sample was a solid tan nodular-appearing tissue measuring 2.0 x 1.5 x 1.0 cm. Biopsy revealed numerous non-caseating epithelioid granulomas present in the lymphoid tissue. Stains were negative for acid-fast bacilli and fungus. The biopsy results were consistent with sarcoidosis. Flow cytometry later showed no evidence of B or T cell lymphoma.

A tentative diagnosis of sarcoidosis was made. The granulomatous disease is seen only in sarcoidosis, tuberculosis, or fungal infections. The non-caseating granulomatous disease in the lymph nodes pointed most closely to sarcoidosis. The pulmonologist considered this to be a case of extrapulmonary sarcoidosis because only hilar nodes were involved without any lung parenchymal involvement. It was thought conceivable that the ANA elevation may have been related to sarcoidosis, although we agreed to continue to monitor the patient for other collagen vascular diseases.

During the hospitalization, the patient had daily high-grade fevers. The fevers were intermittent, daily, variable, and went from 38.4 to a high of 39.4 °C. The patient was also seen by ophthalmology, and she was diagnosed with blepharitis. No uveitis or episcleritis was noted. She was treated with eye drops containing steroids, antimicrobials, and artificial tears. She was started on prednisone 40 mg PO daily with outpatient follow-up. Her fever and ocular symptoms resolved. She was seen in an outpatient clinic prior to the onset of the coronavirus disease 2019 (COVID-19) pandemic and then moved to a different location.

## Discussion

Sarcoidosis is a multisystem granulomatous disease characterized by non-caseating granulomas [1]. It is an uncommon cause of fever of unknown origin [2]. Some definitions of fever of unknown origin include a temperature of more than 38.3 °C (100.9 °F) with an uncertain origin, manifesting on several occasions, and occurring for more than three days of in-hospital stay [2]. Recent literature reviews on the subject tend to categorize causes of fever of unknown origin within broad groupings including infection, inflammatory causes (which would include causes such as sarcoidosis), neoplasia, and miscellaneous and undiagnosed diseases [2]. Clinical algorithms in the literature have emphasized "clue-based" approaches to narrow down the differential to minimize testing, as a fever of unknown origin can potentially include over 200 differential diagnoses; some of the more common of these differentials include lymphoma, tuberculosis, adult Still's disease, and drug fever [3]. However, the literature has reported various exotic cases of fever of unknown origin eventually confirmed as sarcoidosis [4,5].

The clinical presentation of sarcoidosis is variable and can range from an asymptomatic presentation all the way to death due to pulmonary or cardiac involvement [6]. Some features that are more likely in sarcoidosis include renal failure, subcutaneous nodules, uveitis, hepatosplenomegaly, bilateral hilar lymphadenopathy, hypercalcemia, elevated ACE levels, chest X-ray infiltrates, and cardiomyopathy [6]. An elevated ANA prevalence in the setting of sarcoidosis has also been reported in the literature [7].

Sarcoidosis with asymmetric or isolated unilateral lymphadenopathy is unusual and has been reported in only up to 5% of cases of pulmonary sarcoidosis [8]. More typical presentations include bilateral hilar lymphadenopathy. Prior case reports of unilateral hilar lymphadenopathy have confirmed similar delays in diagnosing sarcoidosis based only on biopsy [9]. Up to 60% of systemic sarcoidosis patients show some form of ocular involvement [10]. The most common ocular manifestations of sarcoidosis are uveitis, conjunctival nodules, and dry eyes [11]. Red eye has been reported in sarcoidosis due to "conjunctival involvement" [12].

Our patient's presentation was atypical as sarcoidosis uncommonly presents with unilateral lymphadenopathy and is an uncommon cause of fever of unknown origin. Furthermore, blepharitis is a common and benign ocular presentation that is not as specific for sarcoidosis. This presentation led to delays in narrowing down the diagnosis.

The diagnostic problem resolved eventually, and only on biopsy. Our case highlights the importance of maintaining a heightened suspicion for sarcoidosis in the differential diagnosis of cases involving fever of unknown origin with unilateral hilar lymphadenopathy and ocular manifestations.

## Conclusions

Fever of unknown origin with unilateral hilar lymphadenopathy is a non-classical presentation of sarcoidosis. Blepharitis may also be seen as the presenting ocular manifestation in sarcoidosis in such cases. Considering sarcoidosis early in the differential diagnosis in cases of fever of unknown origin with unilateral lymphadenopathy and ocular manifestations may lead to faster diagnosis, reduced length of stay, and reduced costs of investigations.
